# Challenges in the Diagnosis and Management of Bacterial Lung Infections in Solid Organ Recipients: A Narrative Review

**DOI:** 10.3390/ijms21041221

**Published:** 2020-02-12

**Authors:** Manuela Carugati, Letizia Corinna Morlacchi, Anna Maria Peri, Laura Alagna, Valeria Rossetti, Alessandra Bandera, Andrea Gori, Francesco Blasi, on behalf of the IFALT Working Group

**Affiliations:** 1 Internal Medicine Department, Division of Infectious Diseases, Fondazione IRCCS Cà Granda Ospedale Maggiore Policlinico Milano, 20122 Milano, Italy; anninaperi@gmail.com (A.M.P.); laura.alagna@policlinico.mi.it (L.A.); alessandra.bandera@unimi.it (A.B.); andrea.gori@unimi.it (A.G.); 2 Division of Infectious Diseases and International Health, Duke University, Durham, NC 27710, USA; 3 Internal Medicine Department, Respiratory Unit and Adult Cystic Fibrosis Center, Fondazione IRCCS Cà Granda Ospedale Maggiore Policlinico Milano, 20122 Milano, Italy; letizia.morlacchi@gmail.com (L.C.M.); valeria.rossetti@policlinico.mi.it (V.R.); francesco.blasi@unimi.it (F.B.); 4 Department of Pathophysiology and Transplantation, Università degli Studi di Milano, 20122 Milano, Italy; 5 Centre for Multidisciplinary Research in Health Science, 20122 Milano, Italy

**Keywords:** solid organ transplants, respiratory infections, pneumonia

## Abstract

Respiratory infections pose a significant threat to the success of solid organ transplantation, and the diagnosis and management of these infections are challenging. The current narrative review addressed some of these challenges, based on evidence from the literature published in the last 20 years. Specifically, we focused our attention on (i) the obstacles to an etiologic diagnosis of respiratory infections among solid organ transplant recipients, (ii) the management of bacterial respiratory infections in an era characterized by increased antimicrobial resistance, and (iii) the development of antimicrobial stewardship programs dedicated to solid organ transplant recipients.

## 1. Introduction

In the last decades, the introduction of effective immunosuppressive agents has reduced the risk of rejection of transplanted organs; nonetheless, the long-term immunosuppressive state of solid organ transplant recipients (SOTRs) has increased their susceptibility to infections, especially lung infections. Management of lung infections in SOTRs is complex: the spectrum of pathogens possibly involved is broad, infections can progress rapidly even though signs and symptoms are often mild, and antimicrobial resistance is widespread [1]. In particular, lung infections currently represent a leading cause of morbidity and mortality among SOTRs and threaten the success of transplantation [2].

The severity of lung infections in SOTRs is increased by several factors. First, the rapid and accurate identification of the causative pathogens of lung infections is critical for the appropriate therapeutic management of patients and to avert fatal outcomes. Unfortunately, conventional microbiology is characterized by suboptimal diagnostic accuracy, and few studies have attempted to describe the etiologic distribution of lung infections in SOTRs [3]. Second, the adverse outcomes associated with lung infections in SOTRs have led to the common use of prolonged courses of broad-spectrum antimicrobial agents in SOTRs. Though well intended, overuse of antibiotics contributes to the occurrence of adverse drug effects, drug–drug interactions, prolonged hospitalization, and increased health care costs [4]. Specifically, in SOTRs, this overuse contributes to the development of infections by multidrug-resistant (MDR) Gram-negative pathogens. In several studies performed among solid organ transplant recipients in Western countries, multidrug resistance was frequently encountered in the setting of Gram-negative bloodstream infections [5,6,7,8,9,10]. Of note, a study performed by Linares et al. in Spain reported the detection of extended-spectrum beta-lactamases in 54% of the *Escherichia coli* bacteremia episodes, while Johnson et al. detected the presence of multidrug resistance in 43% of the *Pseudomonas aeruginosa* bloodstream infections reported in a large US transplant center [5,9]. Third, limited data exist about the feasibility and applicability of antimicrobial stewardship (AMS) programs for SOTRs, since many established AMS programs are not inclusive of SOTRs and most studies of antimicrobial stewardship specifically exclude SOTRs. In this review, we discuss the above factors which contribute to the severity of lung infections in SOTRs.

## 2. Results

### 2.1. Etiologic Diagnosis of Lung Infections in Solid Organ Transplant Recipients

Conventional microbiology, which includes classical culture methods, antigen detection methods, serological methods, nucleic acid amplification methods, matrix-assisted laser desorption ionization–time of flight (MALDI-TOF) tests, and other diagnostic tools, provides relevant information for medical practice, allowing the identification and quantification of organisms in clinical samples and increasing the appropriateness of antimicrobial treatments [11].

Despite its undoubted clinical relevance, the performance of conventional microbiology is still suboptimal in many circumstances, especially in the setting of SOTRs, where the rapid and accurate identification of the causative pathogens of respiratory infections is critical for appropriate therapeutic management [3]. The most significant pitfalls of conventional microbiology are described below. 

First, conventional microbiology’s diagnostic capacity is restricted only to known or culturable pathogens. The wide range of potential opportunistic and non-opportunistic pathogens causing lung infections in SOTRs makes direct pathogen identification challenging [12]. However, most diagnostics today still focus on pathogen detection as the reference diagnostic approach. Even recently implemented microbiological diagnostics, such as MALDI-TOF and nucleic acid amplification tests, rely on pathogen detection. The clinical drawbacks of diagnostic approaches limited to known or culturable agents were well depicted by the events of 2009, when existing molecular assays failed to effectively detect a novel pandemic influenza virus. 

Second, current diagnostics have suboptimal sensitivity and specificity. For example, *Candida* infections are frequently encountered among SOTRs, as shown by recent investigations among a high-volume US transplant center [13]. Unfortunately, *Candida* blood culture sensitivity is well known to be <60%, making the diagnosis of *Candida* invasive infections troublesome [14].

Third, while detecting pathogens, current tests are unable to discriminate between pathogens causing active disease and pathogens colonizing asymptomatic carriers. This is particularly relevant in the setting of cystic fibrosis lung transplant recipients. Both in the pretransplant and post-transplant periods, different microbial populations are detected in the airways of cystic fibrosis patients [15,16]. In the case of an acute respiratory infection, the identification of the causative pathogen among the many different organisms retrieved from respiratory cultures is extremely challenging [17].

Fourth, some SOTRs suffer from lung infections due to multiple simultaneous pathogens (such as concomitant influenza and fungal/bacterial pneumonia), where the detection of a single pathogen still omits critical information related to patient care.

Fifth, current diagnostic methods are limited by a prolonged wait for available results, which range from days to weeks, as in the case of mycobacterial cultures. The long turn-around times of nontuberculous mycobacterial cultures are prohibitive for critically ill solid organ transplant recipients, especially in the post-transplant period [18]. Long turn-around times still affect recently introduced diagnostic tests, such as MALDI-TOF. Specifically, while MALDI-TOF allows a more rapid identification of pathogens than biochemical tests, MALDI-TOF can be applied only to pure culture on solid media and not to unprocessed clinical samples. In other words, MALDI-TOF does not eliminate the time needed to obtain a pure culture, which is an important limiting step, especially in the setting of fastidious organisms. Furthermore, while detecting a wide range of bacteria, MALDI-TOF may not be conclusive for less common organisms, such as molds, which play a substantial role as a cause of lung infections in SOTRs. Based on these considerations, MALDI-TOF does not eliminate the need for fungal cultures and their prolonged time for results [19]. 

Histology, specifically transbronchial biopsies, also have several limitations: they are invasive, expensive, subject to sampling error and interobserver variability, and they may cause morbidity. 

Therefore, based on the limitations of microbiology and histology, new diagnostic methods are strongly needed, as recognized by several researchers and international societies, such as the Antibacterial Resistance Leadership Group [3,20]. 

Preliminary studies performed among nontransplant populations showed the potential role of evaluating the host response to respiratory infections. Since immune response is largely conserved for a given pathogen class (e.g., bacteria, viruses, fungi, and protozoa), class-specific host gene response signatures can be identified. Transcriptome signatures are a powerful diagnostic tool in the setting of respiratory infections, as shown by several researchers [21,22,23]. Specifically, Suarez et al. identified a 10-gene classifier to discriminate bacterial and viral lung infections; this classifier was characterized by high sensitivity and specificity (95% and 92%, respectively). Bhattacharya et al. developed an 11-gene signature that discriminated bacterial and nonbacterial lung infections with good diagnostic accuracy (sensitivity of 90% and specificity of 83%). Tsalik et al. designed three separate classifiers in order to differentiate bacterial respiratory infections, viral respiratory infections, and noninfectious conditions. When pooled together, the classifiers designed by Tsalik et al. showed an overall diagnostic accuracy close to 90%. SOTRs would benefit the most from host gene diagnostics, since respiratory infections are among the leading causes of morbidity and mortality in the post-transplant period and SOTRs are exposed to the risk of antibacterial overuse. Discriminating between bacterial and viral infections by the use of transcriptome classifiers such as the ones proposed by Tsalik et al. would reduce inappropriate antibacterial treatments and would hopefully limit the spread of antimicrobial resistance. Further studies are needed to assess the diagnostic validity of transcriptome classifiers among immunocompromised patients, such as SOTRs. 

Finally, despite the abundance of scientific reports describing lung infections in SOTRs, few studies have attempted to evaluate the etiologic distribution of lung infections in this population (Table 1) [24,25,26,27,28,29,30,31,32,33,34,35,36,37,38,39,40,41].

A clear understanding of the etiologic distribution of lung infections in SOTRs is hampered by several factors. First, lung infection studies in SOTRs generally lack a clinical case definition that is both sensitive and specific, which leads to missing a substantial proportion of SOTRs with lung infections or including a significant number of SOTRs without lung infections in etiologic studies. Clinical case definitions are widely used only for nontuberculous mycobacterial and fungal lung infections in SOTRs [42,43]. Second, the accuracy of etiologic studies is limited by rarely testing the tissue where the infection occurs, that is, the lung. The etiology of lung infections in SOTRs is frequently inferred from tests performed on samples contiguous to the pulmonary tissue or from tests performed on body fluids that are a proxy for the lung tissue, such as sputum, nasopharyngeal secretions, blood, or urine. Third, the etiology of lung infections in SOTRs is dynamic and reflects changes in immunosuppressive treatments, antimicrobial prophylaxis, vaccination programs, surgical procedures, and laboratory diagnostic tests. The evolution over time of the etiologic distribution of lung infections in SOTRs poses a restraint on the applicability of etiologic studies performed in the past. Similarly, the selective geographical distribution of certain respiratory pathogens, such as endemic fungi, *Mycobacterium tuberculosis*, or nontuberculous mycobacteria, constitutes an obstacle to the generalizability of etiologic studies. Fourth, the pathophysiology behind single-pathogen and multi-pathogen lung infections in SOTRs has not yet been fully described, making the interpretation of diagnostic tests difficult if not misleading [44]. Fifth, fatal cases are usually under-represented in etiologic studies. This may be due to the fact that (i) the sickest SOTRs often die soon after presentation; (ii) in cases of severe lung infections, resuscitation and medical procedures have priority over research studies; (iii) sick patients may not be candidates for invasive procedures (e.g., a severe lung infection in a SOTR might make a bronchoscopy impractical due to the oxygen requirement of the patient); and (iv) autopsy studies are rarely performed. The under-representation of fatal cases in lung infection studies leads to uncertainty about the causes of lung infection mortality among SOTRs. Sixth, lung infection etiologic studies in SOTRs require analytical approaches that can integrate the results of multiple tests on multiple samples and that can account for the imperfect sensitivity and specificity of each diagnostic test. The scientific literature is rich in case-only studies, where the etiologic distribution of infections is derived from latent class analysis or expert review; similarly, logistic regression and attributable fraction analysis have been applied to case–control etiologic studies. Each of these analytical methods has significant limitations and none integrate multiple test results [45].

The limitations of etiologic research in the setting of lung infections in SOTRs are similar to the limitations perceived in pneumonia etiologic studies. A substantial attempt to overcome the limitations of pneumonia etiologic studies was promoted in 2007 by the Bill & Melinda Gates Foundation and resulted in the international “Pneumonia Etiology Research for Child Health” (PERCH) study [46,47]. PERCH aimed to characterize the etiology of severe pneumonia in children in Sub-Saharan Africa and Asia. To achieve this goal, PERCH enrolled more than 4000 pneumonia cases and more than 5000 controls and is likely the most comprehensive etiologic study of pneumonia. Of note, PERCH not only enrolled a large study population but also developed a Bayesian integrated approach that incorporated evidence from multiple samples from cases and controls and that accounted for the imperfect diagnostic accuracy of the tests used [46,47]. The application of novel analytical methods, such as the PERCH integrated analysis, could be a key component of future studies assessing the etiology of lung infections in SOTRs.

### 2.2. Management of Lung Infections in Solid Organ Transplant Recipients in the Era of Increased Antimicrobial Resistance

Bacterial infections have always been a leading cause of morbidity and mortality among SOTRs, threatening the success of transplantation [2]. In the last decades, the severity of bacterial infections in SOTRs increased due to the emergence of MDR Gram-negative organisms, defined as bacteria non-susceptible to ≥1 agent in three or more antimicrobial categories [48,49,50,51]. Frequent antibiotic exposure, microbiome alterations, recurrent hospitalizations, and hepatic failure are major risk factors associated with MDR Gram-negative infections in the pretransplant period. Renal replacement therapy, prolonged mechanical ventilation, and tracheal intubation in the post-transplant period increase the susceptibility of SOTRs to MDR Gram-negative infections [48]. 

The identification of MDR Gram-negative organisms in SOTRs poses several questions. Once MDR Gram-negative organisms are identified in respiratory samples by microbiological tests, their role in the host still remains to be ascertained. Specifically, MDR Gram-negative organisms could either colonize the airways or they could cause respiratory infections. Airway colonization is characterized by the presence of MDR Gram-negative organisms in the respiratory secretions, detected by microbiological tests in the absence of symptoms, as well as radiologic and endobronchial changes [52]. While MDR Gram-negative organism airway colonization does not warrant treatment, it is a very important risk factor for invasive respiratory infections [53]. Furthermore, in the setting of lung transplantation, MDR Gram-negative organism airway colonization is a known independent determinant of chronic organ dysfunction [54]. Transient bacterial airway colonization can significantly increase bronchoalveolar lavage (BAL) neutrophils and other indicators of lung inflammation [55]. Botha et al. examined 155 consecutive lung transplants and reported that de novo allograft colonization with *P. aeruginosa* was strongly associated with developing bronchiolitis obliterans syndrome (BOS) within 2 years of transplantation [56]. Vos and collaborators reported that persistent *P. aeruginosa* colonization was an even greater risk for BOS than de novo colonization [57,58]. Additionally, Gottlieb et al. found that persistent allograft colonization with *P. aeruginosa* in recipients with cystic fibrosis significantly increased the prevalence of BOS [59].

Among MDR Gram-negative bacteria, *P. aeruginosa, Acinetobacter baumanii*, and carbapenem-resistant Enterobacteriaceae are the main causative agents of respiratory infections in SOTRs. Therapeutic options for the treatment of MDR *P. aeruginosa, A. baumanii*, and carbapenem-resistant Enterobacteriaceae are limited.

MDR *P. aeruginosa* can be responsible for early post-transplant respiratory infections in SOTRs, especially among lung transplant recipients [6,7,60,61]. The higher risk of MDR *P. aeruginosa* respiratory infections among lung transplant recipients compared with other solid organ transplants may be due to the persistent colonization of the upper airways and paranasal sinuses in the pre- and post-transplant periods. In this regard, Walter et al. found that *P. aeruginosa*-free lungs transplanted in cystic fibrosis patients became infected with *P. aeruginosa* clones that were identical in genotype to the isolates from the explanted cystic fibrosis lungs, highlighting that colonization occurred from the patients’ upper airways and sino-nasal space [62].

In the pipeline of new antibiotics, ceftolozane/tazobactam has a major role in the treatment of MDR *P. aeruginosa* infections. Ceftolozane/tazobactam is stable against overexpression of *Pseudomonas*-derived cephalosporinase or efflux pumps as well as against extended-spectrum beta-lactamases, conserving activity against most pan-β-lactam-resistant clinical strains [63]. On the contrary, ceftolozane/tazobactam is susceptible to hydrolysis by carbapenemase enzymes, such as metallo-β-lactamases (MBLs); in such cases, old antibiotics, such as aminoglycosides or colistin, are often the only possible therapeutic options. The use of aztreonam in combination with avibactam for MBLs-producing *P. aeruginosa* might be considered in selected cases of proven susceptibility; however, the production of MBLs in *Pseudomonas* is often associated with other mechanisms of beta-lactam resistance, making this combination not active [64].

Due to its nephrotoxicity, colistin is recommended only in situations where ceftolozane/tazobactam cannot be used [48]. Overall, screening for carbapenemases production as well as susceptibility testing for new antibiotics is highly warranted in order to define the most appropriate treatment for MDR *P. aeruginosa* infections in SOTRs. Finally, recent retrospective studies and meta-analyses did not show any survival benefit in the use of combination regimens compared to monotherapy for the treatment of MDR *P. aeruginosa* [65,66,67,68]. 

Studies investigating the distribution, drug resistance, and clinical characteristics of *A. baumannii* infections in SOTRs reported that more than 80% of such infections were due to MDR or extensively drug resistant (XDR) strains, with almost all of these isolates being resistant to carbapenems and an overall infection-related mortality up to 40% [69,70]. 

No new antibiotics are currently available for the treatment of MDR *A. baumannii* and clinicians should rely on combinations of old antimicrobials. Antibiotics for MDR *A. baumannii* include polymyxins, ampicillin/sulbactam, and tigecycline. Rifampicin, glycopeptides, or fosfomycin can be used in association with other active molecules, such as polymyxins. Susceptibility testing should always be performed in order to allow the choice of an appropriate therapy according to the specific isolate.

Colistin demonstrated its activity against MDR *A. baumannii* in several retrospective studies, despite a worrisome risk of selection of resistances when used in monotherapy [71,72,73]. In a study by Shields et al., treatment with a colistin/carbapenem regimen was an independent predictor of 28-day survival in SOTRs with XDR *A. baumannii* respiratory infections [74]. While in vitro synergism against *A. baumannii* was described for colistin and rifampin, no differences in mortality were found in patients with *A. baumannii* pneumonia treated with colistin and rifampin compared to patients treated with colistin monotherapy [75,76]. Furthermore, the use of rifampicin in SOTRs is limited by the pharmacological interactions of rifampin with several immunosuppressive drugs, including tacrolimus and cyclosporine. Sulbactam retains intrinsic activity against *A. baumannii*, and its association with ampicillin has been demonstrated to be effective against severe infections due to *A. baumannii*, including pneumonia [77]; however, mortality in this setting remains high and resistances to this combination are rising [78]. Tigecycline also retains in vitro activity against *A. baumannii*. Unfortunately, tigecycline use is not recommended in the setting of respiratory infections due to tigecycline pharmacokinetic characteristics. Tigecycline may be an option for the treatment of *A. baumannii* pneumonia only if the MIC is ≤1 mg/L and the isolate is resistant to other agents. In this event, high doses (loading dose of 200 mg followed by 100 mg every 12 h) are preferable [79]. Eravacycline may represent a future option for the treatment of MDR *A. baumannii* in SOTRs.

SOTRs are at increased risk for carbapenem-resistant Enterobacteriaceae infections, especially carbapenem-resistant *Klebsiella pneumoniae* infections [80]. Giannella et al. described 20 carbapenem-resistant *K. pneumoniae* infections among 237 liver transplant recipients, with respiratory infections accounting for 15.0% of the carbapenem-resistant Enterobacteriaceae infections. Infections were more common among carbapenem-resistant *K. pneumoniae*-colonized patients than among non-colonized patients [81]. Similar results were reported by Bergamasco et al. [82].

Different new antibiotics have recently become available for carbapenem-resistant Enterobacteriaceae. However, the experience with these molecules in real-life settings is still scarce, as is the evidence of their superiority versus old schemes [83,84,85]. Data are even more scarce in the setting of SOTRs, as they are mainly limited to small series rather than large cohorts [10,86,87,88]. Ceftazidime/avibactam inhibits the activity of class A and D carbapenemases, including KPC and OXA 48, but it is not active against class B carbapenemases. Ceftazidime/avibactam is approved for hospital-acquired and ventilator-associated pneumonia, although recent studies recognized pneumonia as a risk factor for microbiologic failure and ceftazidime/avibactam resistance among patients with carbapenem-resistant Enterobacteriaceae infections [89]. Although there is no evidence to support recommending the use of ceftazidime/avibactam in association with other antimicrobials, combination treatment seems strongly reasonable given the recent report of resistances and the high mortality associated with infections due to carbapenem-resistant Enterobacteriaceae [89]. Other new antibiotics likely to have a role per se or within combination therapies in the treatment of carbapenem-resistant Enterobacteriaceae infections include cefiderocol, meropenem/vaborbactam, imipenem/relebactam, and plazomicin [90,91,92].

### 2.3. Antimicrobial Stewardship Programs in Solid Organ Transplant Recipients in the Era of Increased Antimicrobal Resistance

AMS programs are crucial to counteract antimicrobial resistance. As suggested by the US Centers for Disease Control and Prevention (CDC), AMS programs should work on key drivers for reducing inappropriate antimicrobial utilization. The following interventions were proposed by the US CDC as key drivers: (i) promoting a culture of optimal antibiotic use within a facility, (ii) timely and appropriate initiation of antibiotics, (iii) appropriate administration and de-escalation, and (iv) favoring AMS program data monitoring and transparency and creating a stewardship infrastructure [93]. 

Despite the US CDC recommendations, AMS programs are still uncommon among immunocompromised patients, and SOTRs specifically. A large survey conducted in 2015 in 71 US transplant centers described the presence of an AMS program in 62 of the 71 centers surveyed (87%). Of the 62 AMS programs, the proportion performing stewardship activities that were inclusive of adult and pediatric SOTRs was 46 (74%) and 29 (47%), respectively. This survey also found that commonly developed institutional guidelines for SOTRs were focused on management of invasive fungal infections, CMV infection, and fever and neutropenia [94]. Institutional guidelines did not provide guidance for the use of antibacterials in SOTRs. Furthermore, concordance of anti-infective treatment prescriptions with available recommendations and guidelines was suboptimal among SOTRs. One of the largest Canadian transplant centers conducted real-time audits on all antimicrobial therapies in transplant patients in 2013 and assessed each regimen against stewardship principles established by the Centers for Disease Prevention and Control, supplemented by applicable transplant-specific infection guidelines. Fifty-eight percent of the transplant recipients audited (103/176) received at least one antimicrobial, of which 30% were discordant with stewardship principles. The most common stewardship-discordant categories were lack of de-escalation (34.4%), empiric antimicrobial spectrum being too broad (12.5%), and therapy duration being too long (21.8%). All these prescription patterns are associated with an increased risk of antimicrobial resistance development. Infectious disease consultation was associated with more stewardship-concordant prescriptions (*p* = 0.03) [4].

Preliminary efforts of AMS interventions in immunocompromised patients have been associated with a positive effect on the outcome of these critically ill patients [95,96]. A large single-center study conducted on SOTRs at Toronto General Hospital demonstrated that formal consultation with an infectious diseases specialist was associated with a significant reduction in 28-day mortality and lower readmission rates, suggesting a clinical benefit from a hospital policy of routine infectious disease specialist consultation in SOT centers [95]. Formal studies assessing the benefits associated with the implementation of AMS programs among SOTRs are urgently needed, as requested in 2013 by some of the leading US transplant centers [97].

## 3. Discussion

This review highlights the many challenges posed by the management of respiratory infections among SOTRs and paves the way for new research studies. Specifically, this review identifies the intrinsic limitations of diagnostic tests, the imperfect knowledge of the etiologic distribution of respiratory infections among SOTRs, the increasing prevalence of MDR Gram-negative pathogens, and the lack of SOTR-dedicated antimicrobial stewardship programs as the main obstacles clinicians face when dealing with respiratory infections in SOTRs.

This review is not exhaustive of all the challenges associated with the diagnosis and management of respiratory infections in SOTRs. For example, while describing the emergence of antimicrobial resistance against bacteria, this review does not cover the issue of resistance among antivirals and antifungals. Similarly, this review does not address in detail opportunistic infections, as well as tuberculous and non-tuberculous mycobacterial respiratory infections. The review does not describe prophylaxis or infection control policies for the containment of respiratory infections among SOTRs.

Despite its limitations, this review calls for innovative epidemiology- and microbiology-based strategies in order to increase survival among solid organ transplant recipients experiencing respiratory infections. Specifically, this review prompts future studies to (i) explore new diagnostic tools, such as host gene expression and microbiome analysis, for identifying the etiologic agents of respiratory infections in SOTRs; (ii) develop accurate analytic methods for describing the etiologic distribution of respiratory infections in SOTRs; (iii) assess the clinical outcomes associated with the use of new antibacterials for respiratory infections in SOTRs; and (iv) evaluate the safety and cost-effectiveness of SOTR-dedicated antimicrobial stewardship programs. 

## 4. Materials and Methods 

An extensive search was conducted of the relevant literature, with a focus on literature published in the last 10 years (2000–2019). Key search concepts used were transplant, solid organ, and bacterial infections. Searches that combined the above search concepts using AND operators were executed using PubMed. The searches were last performed on 20 September 2019.

## Figures and Tables

**Table 1:** Main studies addressing the etiology of lung infections in solid organ transplant recipients.

First Author and Publication Year	Study Population	Study Population Details	Study Design	Study Period	Lung Infections Evaluated	Prevalence of Lung Infections	Diagnostic Tests Implemented	Patients with an Etiological Diagnosis among Patients with Lung Infections	Most Frequently Identified Pathogens	Analytical Methods Used for Etiology Attribution	Reference
Golfieri R, 2000	Liver transplant recipients (n = 300)	Consecutive transplant recipients	Single-center, observational retrospective cohort study	1986–1997	Bacterial, mycobacterial, viral, and fungal lung infections	41/300 (13.7%)	Respiratory cultures	NA	*Pseudomonas aeruginosa*, CMV, *Candida* spp.	Expert review	[24]
Rao KH, 2002	Kidney transplant recipients (n = 40)	Consecutive transplant recipients	Single-center, observational retrospective cohort study	1998–2000	Bacterial, mycobacterial, and fungal lung infections	NA	Respiratory cultures, histology	NA	*Aspergillus spp.*, *Candida spp.*, *Nocardia* spp.	Expert review	[25]
Loinaz C, 2003	Intestinal and multivisceral transplant recipients (n = 124)	Consecutive transplant recipients	Single-center, observational retrospective cohort study	1994–2001	Bacterial lung infections	38/124 (30.6%)	Respiratory cultures	NA	*Pseudomonas aeruginosa*	Expert review	[26]
Bonvillain RW, 2007	Lung transplant recipients (n = 120)	Nonconsecutive bilateral lung transplant recipients	Single-center, observational retrospective cohort study	1990–2005	Bacterial, mycobacterial, viral, and fungal lung infections	NA	Respiratory cultures, blood cultures	NA	*Pseudomonas aeruginosa*, *Staphylococcus aureus*, *Aspergillus* spp.	Expert review	[27]
Husain S, 2007	Lung transplant recipients (n = 116)	Consecutive lung transplant recipients undergoing bronchoscopy	Single-center, observational prospective cohort study	2003–2005	*Aspergillus* infections	6/116 (5.2%)	Respiratory cultures	6/6 (100%)	*Aspergillus* spp.	Expert review	[28]
Campos S, 2008	Lung transplant recipients (n = 49)	Consecutive transplant recipients	Single-center, observational retrospective cohort study	2003–2007	Bacterial and fungal lung infections	NA	Respiratory cultures, blood cultures, and histology	NA	*Pseudomonas aeruginosa*, *Staphylococcus aureus*, *Aspergillus* spp.	Expert review	[29]
Mota PC, 2009	Kidney transplant recipients (n = 36)	Consecutive kidney transplant recipients presenting with respiratory symptoms	Single-center, observational retrospective cohort study	NA	Bacterial and fungal lung infections	36/36 (100%)	Respiratory cultures and blood cultures	7/36 (19.4%)	*Streptococcus pneumoniae*, *Hemophilus influenzae*, *Staphylococcus aureus*	Expert review	[30]
Kupeli E, 2011	Kidney transplant recipients (n = 136)	Consecutive kidney transplant recipients	Single-center, observational retrospective cohort study	2007–2010	Bacterial, mycobacterial, and fungal lung infections	12/136 (8.8%)	Respiratory cultures	5/12 (41.7%)	*Streptococcus pneumoniae*, *Acinetobacter baumanii*, *Mycobacterium tuberculosis*	Expert review	[31]
Qin J, 2012	Liver transplant recipients (n = 2550)	Consecutive liver transplant recipients	Single-center, observational retrospective cohort study	2000–2011	Bacterial, mycobacterial, viral, and fungal lung infections	453/2550 (17.8%)	Respiratory cultures, blood cultures, histology, CMV antigenemia	NA	*Staphylococcus aureus*, CMV, *Pseudomonas aeruginosa*	Expert review	[32]
Eyuboglu FO, 2013	Liver, heart, and kidney transplant recipients (n = 998)	HIV-Ab negative consecutive transplant recipients	Single-center, observational retrospective cohort study	2000–2012	Bacterial, mycobacterial, and fungal lung infections	73/998 (7.3%)	Respiratory cultures	32/73 (43.8%)	*Mycobacterium tuberculosis*, *Staphylococcus aureus*, *Moraxella catharralis*	Expert review	[33]
Kim SY, 2013	Lung transplant recipients (n = 48)	Consecutive lung transplant recipients presenting with respiratory symptoms	Single-center, observational retrospective cohort study	NA	Bacterial, mycobacterial, viral, and fungal lung infections	48/48 (100%)	NA	42/48 (87.5%)	*Acinetobacter baumanii*, *Pseudomonas aeruginosa*, *Staphylococcus aureus*	Expert review	[34]
Hekimoglu K, 2015	Liver transplant recipients(n = 188)	Consecutive adult liver transplant recipients presenting with respiratory complications	Single-center, observational retrospective cohort study	2002–2013	Bacterial, mycobacterial, viral, and fungal lung infections	34/188 (18.1%)	NA	34/34 (100%)	*Streptococcus pneumoniae*, *Klebsiella pneumoniae*, *Candida* spp.	Expert review	[35]
Li JJ, 2015	Kidney transplant recipients (n = 52)	Consecutive kidney transplant recipients with respiratory infections	Single-center, observational retrospective cohort study	2008–2013	Bacterial, mycobacterial, viral, and fungal lung infections	52/52 (100%)	Respiratory cultures, blood cultures, NAAT, Aspergillus Ag, 1-3 B dextran antigen	40/52 (46.9%)	Gram-negative bacteria	Expert review	[36]
Shah SK, 2016	Lung transplant recipients (n = 202)	Consecutive lung transplant recipients	Single-center, observational retrospective cohort study	1990–2005	Mycobacterial lung infections	30/202 (14.8%)	BAL, sputum, and blood mycobacterial cultures	NA	*Mycobacterium abscessus*, *Mycobacterium avium*, *Mycobacterium gordonae*.	Expert review	[37]
Aspelund AS, 2018	Lung transplant recipients (n = 126)	Consecutive adult lung transplant recipients	Multicenter, observational prospective cohort study	2012–2014	Bacterial, mycobacterial, viral, and fungal lung infections	57/126 (45.2%)	Respiratory cultures, NAAT, histology	57/57 (100%)	*Candida spp.*, *Pseudomonas aeruginosa*, *Staphylococcus aureus*	Expert review	[38]
Magnusson J, 2018	Lung transplant recipients (n = 98)	Consecutive adult lung transplant recipients	Single-center, observational prospective cohort study	2009–2012	Viral infections	51/98 (52.0%)	NAAT	51/51 (100%)	Rhinovirus, coronavirus, respiratory syncytial virus	Expert review	[39]
Onyearugbulem C, 2018	Lung transplant recipients (n = 98)	Consecutive pediatric lung transplant recipients	Single-center, observational retrospective cohort study	2009–2016	Bacterial, mycobacterial, viral, and fungal lung infections	6/98 (6.1%)	Respiratory cultures, blood cultures, NAAT	6/6 (100%)	Respiratory viruses, *Mycobacterium abscessus*	Expert review	[40]
Tachibana K, 2018	Lung transplant recipients (n = 240)	Consecutive lung transplant recipients	Multicenter, observational retrospective cohort study	2000–2014	Mycobacterial and fungal infections	NA	Respiratory cultures, Aspergillus antigen and antibodies	12/NA	*Mycobacterium avium complex, Mycobacterium fortuitum, Mycobacterium kansasii, Mycobacterium gordonae, Mycobacterium abscessus*, *Aspergillus* spp.	Expert review	[41]

## Abbreviations

AMS	Antimicrobial stewardship
BAL	Bronchoalveolar lavage
CMV	Cytomegalovirus
IFALT	Investigating respiratory FAilure in Lung Transplant patients (IFALT)
MBL	Metallo-β-lactamases
NAAT	Nucleic acid amplification test
SOTR	Solid organ transplant recipients
